# Supplementary material for: Mechanistic models of asymmetric hand-over-hand translocation and nucleosome navigation by CMG helicase
Source: Nat Commun. 2025 Nov 21;16:10304. doi: 10.1038/s41467-025-65232-x (PMC12639105; doi:10.1038/s41467-025-65232-x)
Supplement: Supplementary file 1 — Supplementary Information [file 41467_2025_65232_MOESM1_ESM.pdf]

Supplementary Figures

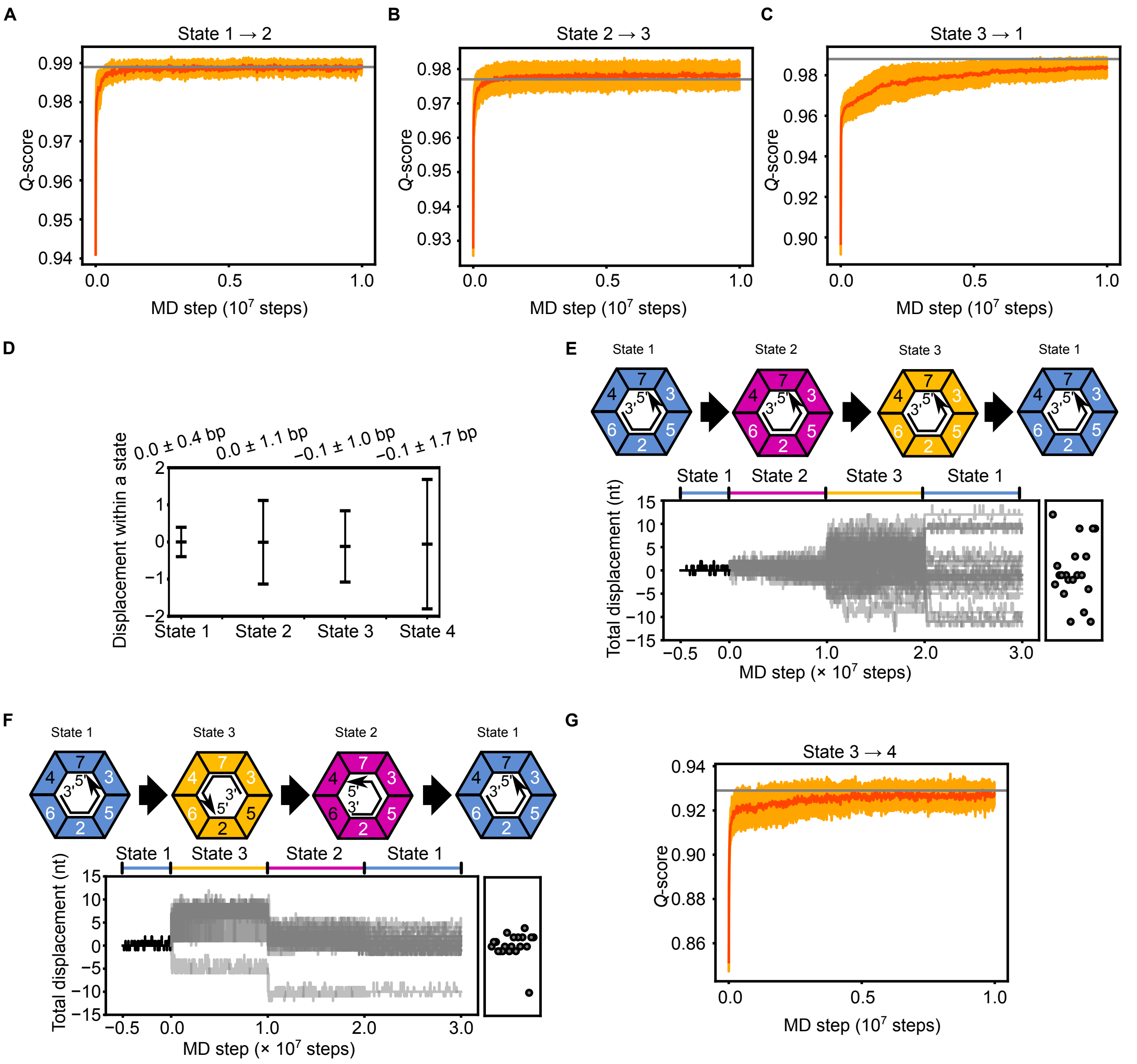

Supplementary Figure 1: Coarse-grained molecular dynamics simulations of CMG on ssDNA using the potential switching approach. (A−C, G) Recovery of Q-score at the transitions of State1→2 (A), State2→3 (B), State3→1 (C), and State3→4 (G). Q-score is defined as the ratio of the number of residue-residue contacts in simulations to the number of natively formed contacts in the reference structure. Red lines and orange areas represent mean ± standard deviation (n = 20). Gray lines represent the equilibrated Q-scores of the State1 (A), State2 (B), State 3 (C), and State4 (G). **(D)** Displacement of CMG within each state (State1–4) under the asymmetric hand-over-hand model. Error bars indicate mean ± standard deviation over 100 frames before potential switching (n = 100). A threshold of ±3 nt (twice the maximum intra-state deviation) was used to classify trajectories as forward (> +3 nt), backward (< −3 nt), or idling (−3 to +3 nt). **(E)** Schematic of the State1→2→3→1 switching scheme without switching hydrogen-bond potentials (top) and representative displacement

trajectories (bottom). The black line denotes the trajectory from State1 used for simulation initiation. The gray lines represent all trajectories (*n* = 20). **(F)** Schematic of the State1→3→2→1 switching scheme (top) and representative displacement trajectories (bottom). The black line denotes the trajectory from State1 used for simulation initiation. The grey lines represent all trajectories (*n* = 20).

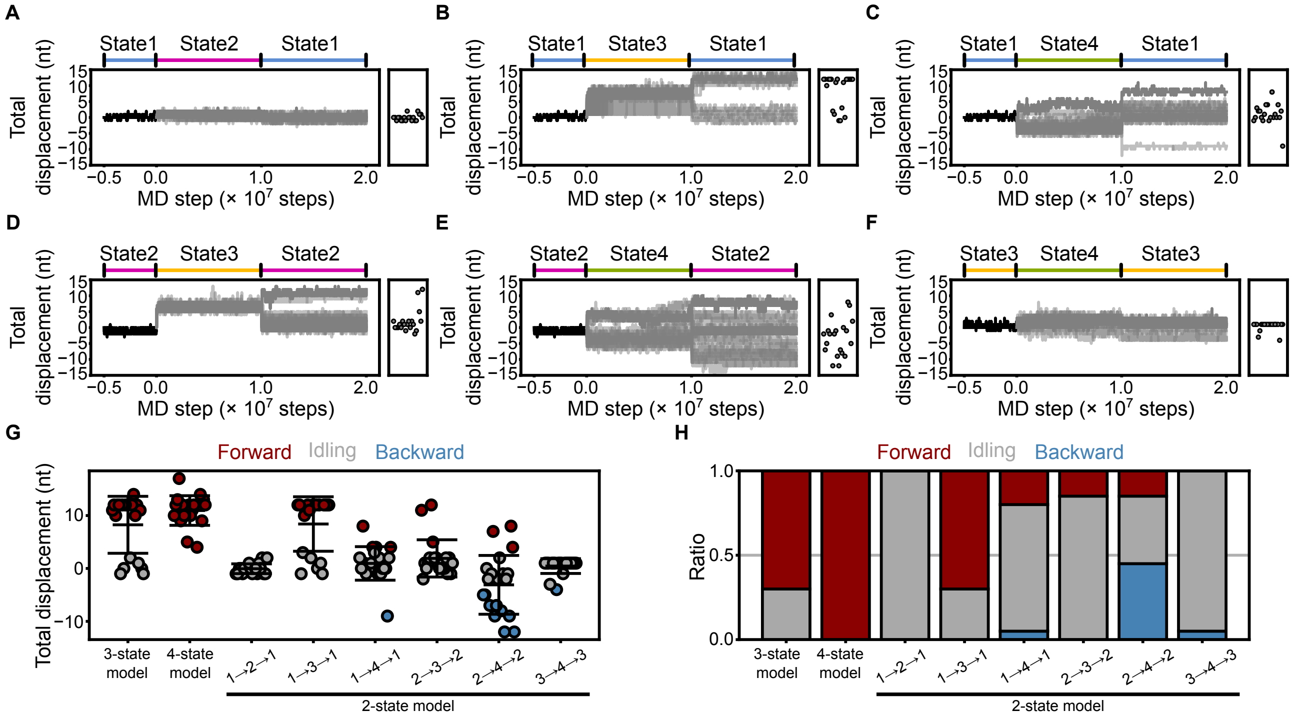

Supplementary Figure 2: Coarse-grained molecular dynamics simulations of CMG on ssDNA using the potential switching between two states (A–F) Time trajectories of CMG displacement under two-state switching schemes: (A) State1→2→1, (B) State1→3→1, (C) State1→4→1, (D) State2→3→2, (E) State2→4→2, and (F) State3→4→3. Black lines denote trajectories used to initiate simulations. The gray lines represent all trajectories. (n = 20). **(G, H)** Final displacement values (G) and trajectory classifications (H) for the three-state, four-state, and various two-state switching models. Red, blue, and grey indicate forward, backward, and idling trajectories, respectively. Error bars in (G) represent mean ± standard deviation (n = 20).

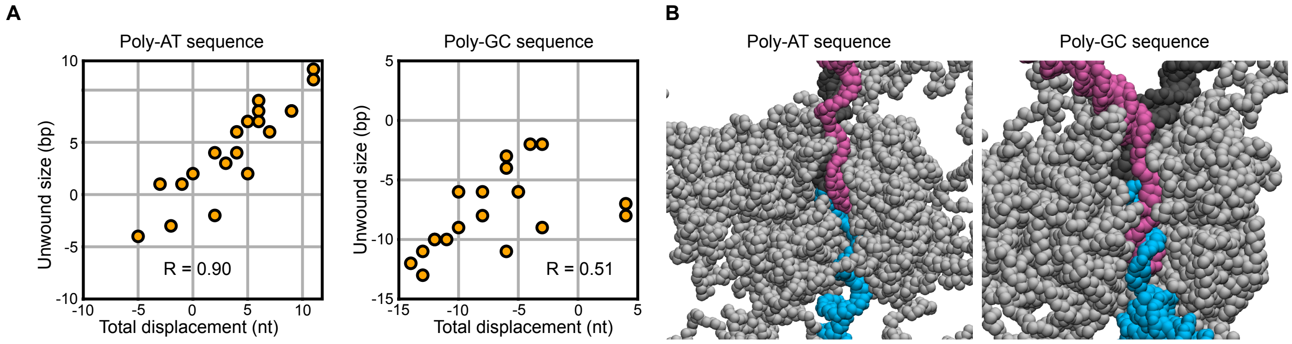

Supplementary Figure 3: Sequence-dependent effects on CMG-mediated dsDNA unwinding. (A) Correlation between final CMG displacement and the number of base pairs unwound for poly-AT (left) and poly-GC (right) DNA sequences (*n* = 20 for both conditions). Pearson's correlation coefficients are shown in each panel. **(B)** Representative snapshots showing the stacking of the lagging-strand ssDNA within the central pore of CMG in simulations with poly-AT (left) and poly-GC (right) DNA substrates. DNA strands are colored as in previous figures.

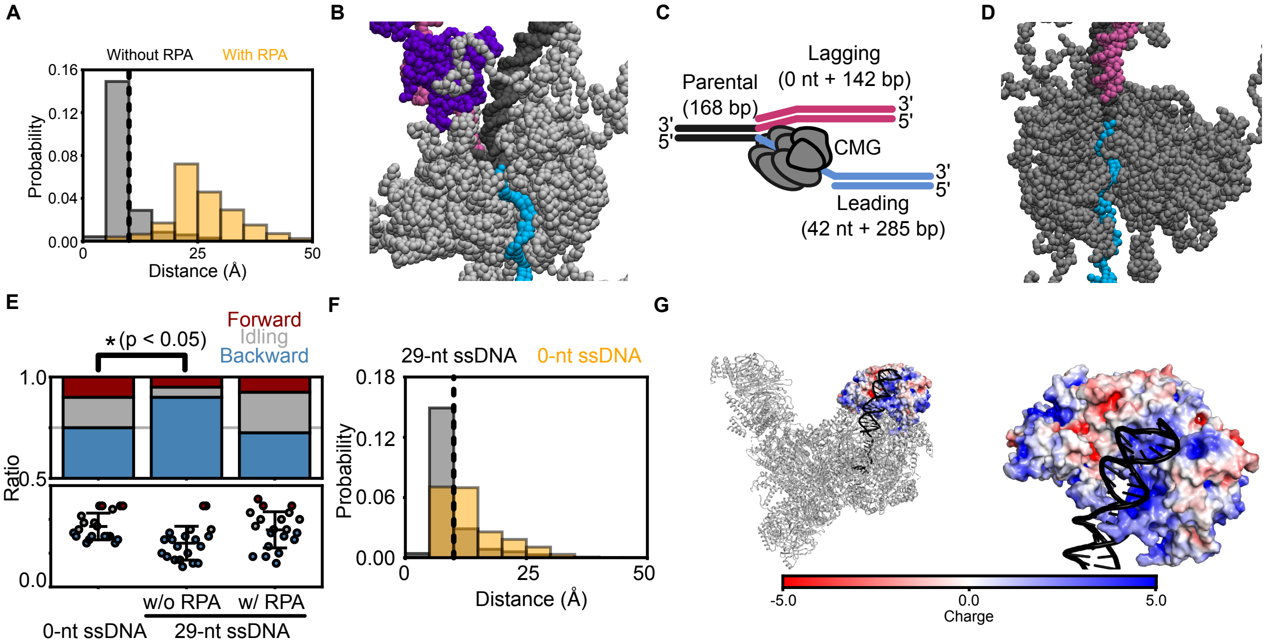

Supplementary Figure 4: Roles of RPA, FPC (Mrc1/Tof1/Csm3), and Ctf4 in CMG-mediated dsDNA unwinding. (A) Distribution of the minimum distance between beads of the lagging-strand poly-GC DNA and residues forming the CMG central pore, with and without RPA. The dashed line at 10 Å indicates the threshold used to define clogging. **(B)** Representative snapshot showing ejection of the lagging-strand DNA from the CMG pore in the presence of RPA. **(C–D)** Initial model of CMG bound to Y-forked DNA where the lagging strand ssDNA was replaced by dsDNA. **(C)** schematic diagram; **(D)** Coarse-grained representation. DNA strands are colored as follows: parental (black), leading (cyan), lagging (magenta); CMG is shown in grey. Mcm7 is omitted to visualize the path of the leading-strand ssDNA through the Mcm2–7 ring. **(E)** Trajectory classification (top) and final displacements (bottom) under poly-GC Y-fork without the lagging-strand ssDNA or with the 29-nt lagging-strand ssDNA. *P* values were calculated using the one-tailed Wilcoxon–Mann–Whitney test [P = 0.001 (Cohen's d = 1.10)]. Error bars represent mean ± standard deviation (n = 20). **(F)** Distribution of the minimum distance between beads of the lagging-strand poly-GC DNA and residues forming the CMG central pore, with and without the lagging-strand ssDNA. The dashed line at 10 Å indicates the threshold used to define clogging. **(G)** Electrostatic surface potential of the Tof1/Csm3 complex from the cryo-EM structure (PDB ID: 8KG6). Left: full complex; right: magnified view of the DNA-binding surface. The potential scale ranges from −5.0 (red) to +5.0 (blue).

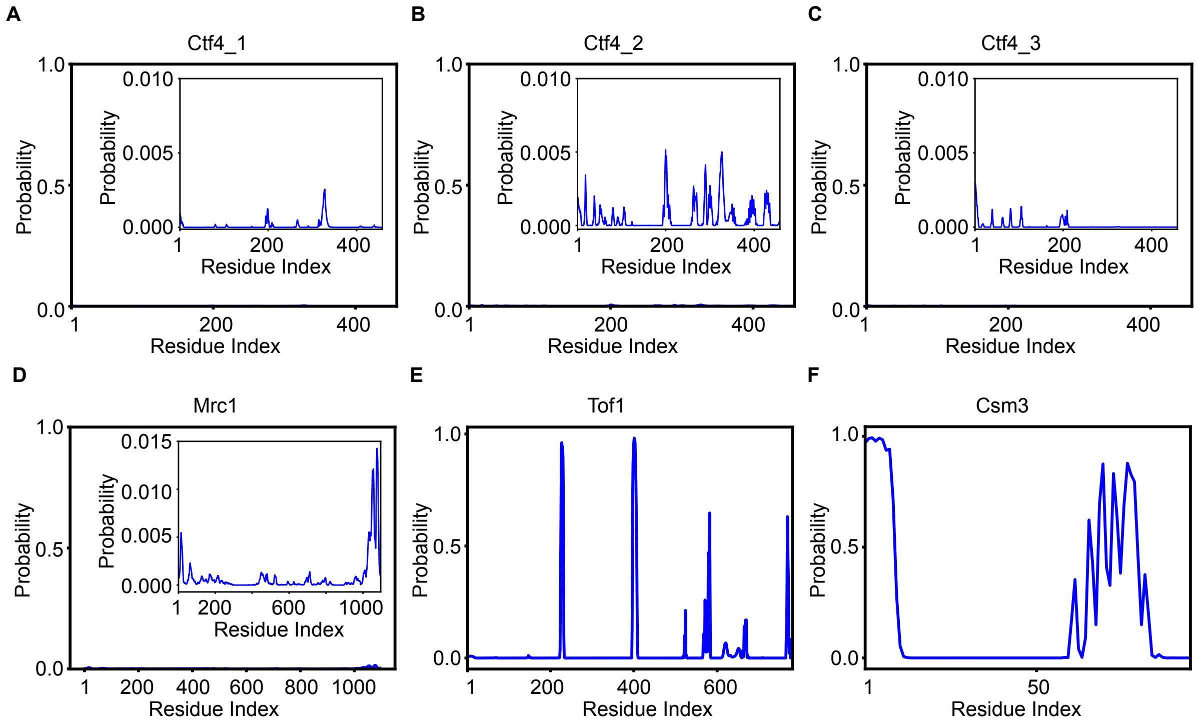

Supplementary Figure 5: Interaction analysis between FPC/Ctf4 and DNA. (A−F) Probability of each residue in either monomer of Ctf4 trimer (A−C), Mrc1 (D), Tof1 (E) and Csm3 (F) contacting DNA. The inset figures in (A−D) were magnified view of the plots.

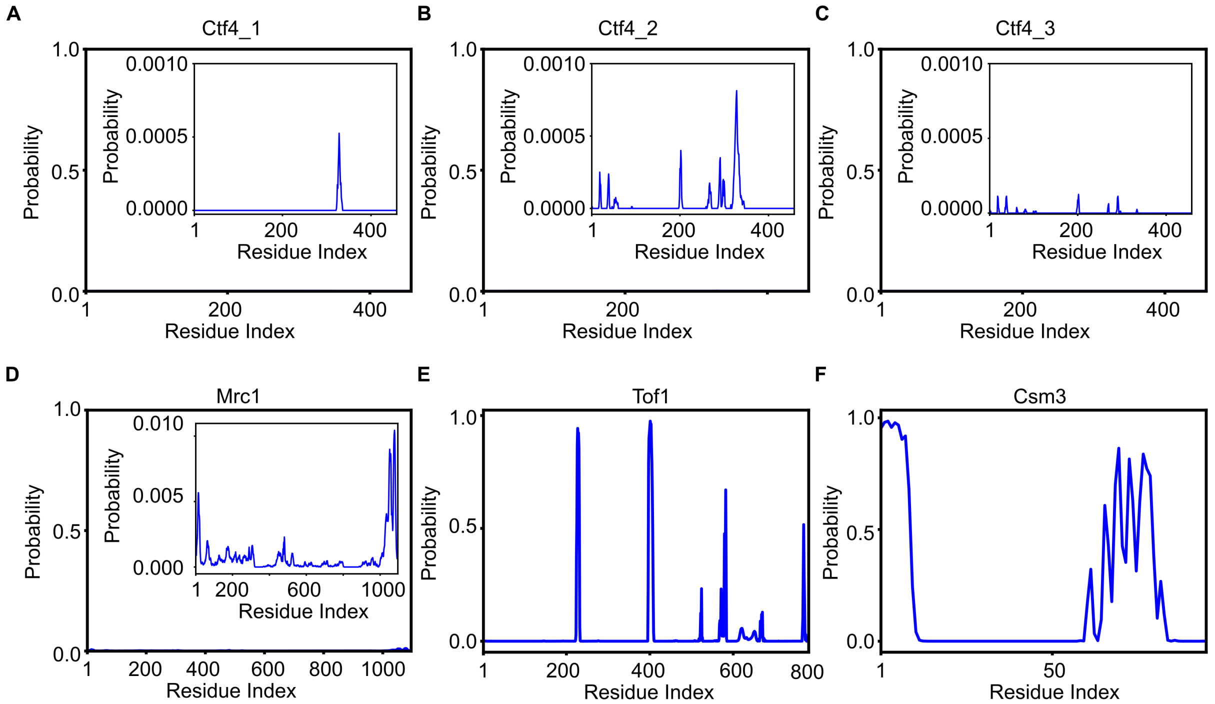

Supplementary Figure 6: Interaction analysis between FPC/Ctf4 and nucleosomal DNA. (A−F) Probability of each residue in either monomer of Ctf4 trimer (A−C), Mrc1 (D), Tof1 (E) and Csm3 (F) contacting nucleosomal DNA. The inset figures in (A−D) were magnified view of the plots.

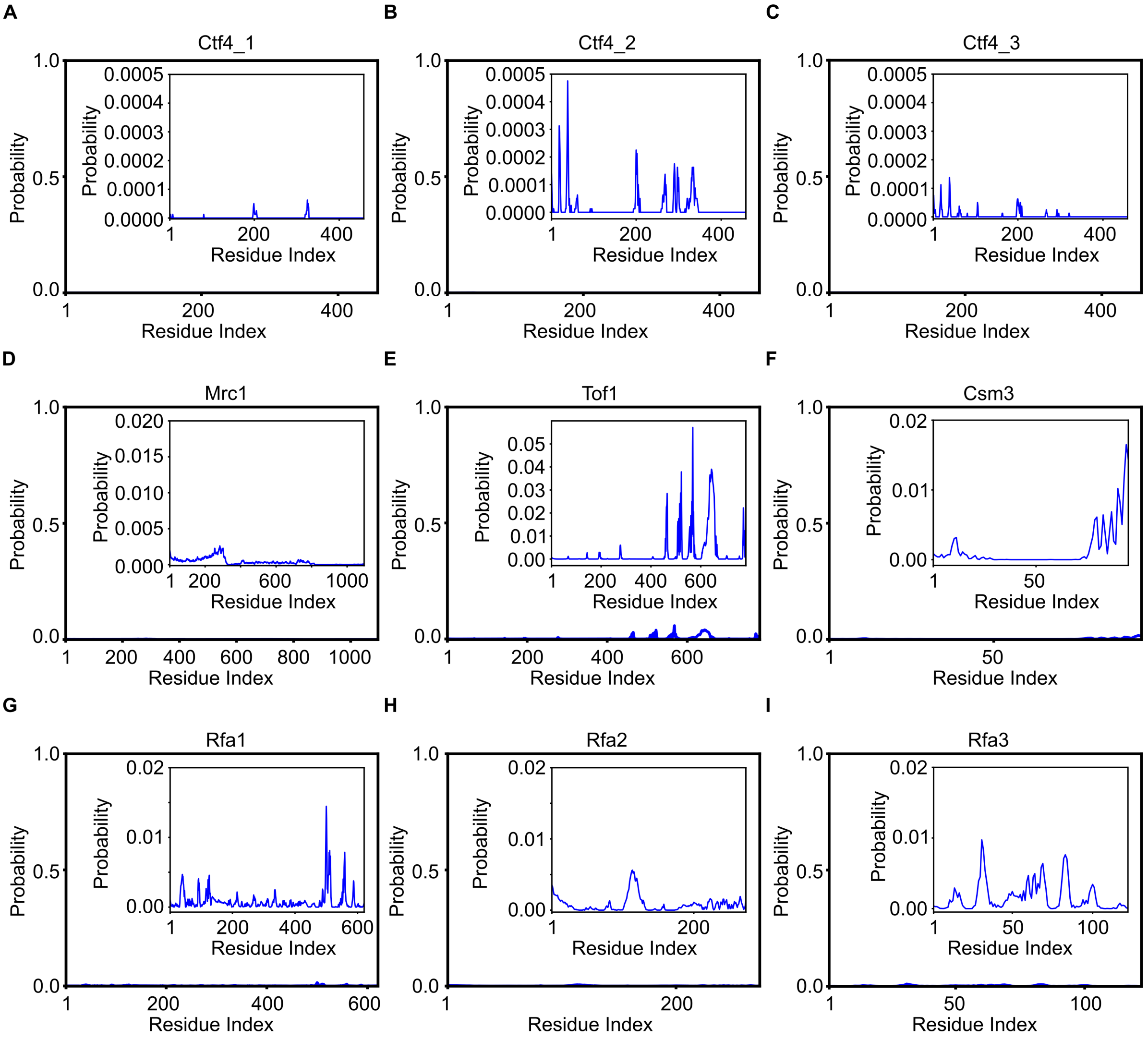

Supplementary Figure 7: Interaction analysis between FPC/Ctf4/RPA and histones. (A−I) Probability of each residue in either monomer of Ctf4 trimer (A−C), Mrc1 (D), Tof1 (E), Csm3 (F), Rfa1 (G), Rfa2 (H), and Rfa3 (I) contacting histones. The inset figures were magnified view of the plots.

Supplementary Table

**Reliability and reproducibility checklist for molecular dynamics simulations**<br>***All boxes must be marked YES by acceptance unless an N/A option is available**	**Yes**	**N/A**	**Response**<br>**(Please state where this information can be found in the text)**
**1. Convergence of simulations and analysis**			
1a. Is an evaluation presented in the text to show that the property being measured has equilibrated in the simulations<br>(*e.g.* time-course analysis)?	☒		The relaxation and equilibration of configuration were evaluated based on Q-score in Supplementary Fig. 1A–C & 1G.
1b. Then, is it described in the text how simulations are split into equilibration and production runs and how much data were analyzed from production runs?	☒		The displacement was calculated from the final frame of md simulations (‘final displacement’ in the text). The extent of nucleosome unwrapping was calculated from the last 100 frames of the simulations.
1c. Are there at least 3 simulations per simulation condition with statistical analysis?	☒		We performed 20 individual runs for each.
1d. Is evidence provided in the text that the simulation results presented are independent of initial configuration?	☒		We individually performed potential switching simulations with different random seeds. This ensures that a distinct configuration undergoes state transition for each trajectory.
**2. Connection to experiments**			
2a. Are calculations provided that can connect to experiments (*e.g.* loss or gain in function from mutagenesis, binding assays, NMR chemical shifts, J-couplings, SAXS curves, interaction distances or FRET distances, structure	☒		Final displacement along ssDNA was consistent with the prediction from Cryo-EM structures. Roles of RPA and dsDNA

factors, diffusion coefficients, bulk modulus and other mechanical properties, *etc*.)?			on the lagging strand in DNA unwinding were also consistent with the previous study. The DNA binding of Csm3/Tof1 was consistent with Cryo-EM structure. The nucleosome function as an energy barrier against CMG is consistent with previous reports.
**3. Method choice**			
3a. Is it described in the text what force field and water model are used and why?	☒		We described the force fields and stated that we used implicit solvent treatment to speed up the simulations in Methods section.
3b. Do simulations contain membranes, membrane proteins, intrinsically disordered proteins, glycans, nucleic acids, polymers, or cryptic ligand binding?	☒	☐	**Our simulations contain nucleic acids.**
If 3b is **YES**, are enhanced sampling methods used?	☐	Response not needed if **N/A**	Response not needed if **N/A**
If enhanced sampling methods are used, are the convergence criteria clearly stated?	☐		
If 3b is **YES**, is it explained in the text why or why not enhanced sampling methods are used?	☒		The coarse-grained model, 3SPN.2C can reproduce sequence-dependent curvature, bending, and melting within simulation timescale.
**4. Code and reproducibility**			
4a. Is a table provided describing the system setup, such as simulation box dimensions, total number of atoms, total	☒		The MD set-up is stated in the Methods section.

number of water molecules, salt concentration, lipid composition (number of molecules and type)?			
4b. Is it described in the text what simulation and analysis software and which versions are used?	☒		The software used in this study is described in the Methods section.
4c. Are initial coordinate and simulation input files and a coordinate file of the final output provided as supplementary files or in a public repository?	☒		The simulation trajectories and input files for running simulations have been submitted to the Biological Structure Model Archive (BSM-Arc) under BSM-ID BSM00082 (10.51093/bsm-00082).
4d. Is there custom code or custom force field parameters?	☐	☒	Response not needed if **N/A**
If **YES**, are they provided as supplementary profiles or in a public repository?	☐		
