# Supplementary material for: Mechanistic models of asymmetric hand-over-hand translocation and nucleosome navigation by CMG helicase
Source: Nat Commun. 2025 Nov 21;16:10304. doi: 10.1038/s41467-025-65232-x (PMC12639105; doi:10.1038/s41467-025-65232-x)
Supplement: Supplementary file 2 — Description of Additional Supplementary Files [file 41467_2025_65232_MOESM2_ESM.pdf]

Description of Additional Supplementary Files

Supplementary Movie 1: The representative trajectory in which CMG translocated along ssDNA by switching potentials among the state 1, 2, and 3. The black and gray beads represent ssDNA and CMG.

Supplementary Movie 2: The representative trajectory in which CMG stepped back at the transition from the state 3 to 1.

Supplementary Movie 3: The representative trajectory in which CMG translocated along ssDNA by switching potential among the state 1, 2, 3, and 4.

Supplementary Movie 4: The representative trajectory in which CMG unwound dsDNA. The black, cyan, and magenta beads represent the parental, leading, and lagging strand DNA.

Supplementary Movie 5: The representative trajectory in which CMG with RPA on the lagging strand unwound dsDNA. The purple beads represent RPA.

Supplementary Movie 6: The representative trajectory in which CMG with Mrc1, Tof1, Csm3, and Ctf4 unwound dsDNA. The green and ice-blue beads represent Mrc1/Tof1/Csm3 and Ctf4.

Supplementary Movie 7: The representative trajectory in which CMG with Mrc1/Tof1/Csm3/Ctf4 and RPA unwound dsDNA.

Supplementary Movie 8: The representative trajectory in which CMG with Mrc1/Tof1/Csm3/Ctf4 and RPA collided with a nucleosome and unwound dsDNA. The blue, red, and yellow beads represent H3/H4, H2A, and H2B.

Supplementary Movie 9: The representative trajectory in which CMG with Mrc1/Tof1/Csm3/Ctf4 and RPA collided with a nucleosome which is partially unwrapped to SHL (–5).

Supplementary Movie 10: The representative trajectory in which CMG with Mrc1/Tof1/Csm3/Ctf4 and RPA collided with a nucleosome which is partially unwrapped to SHL (–4).

Supplementary Movie 11: The representative trajectory in which CMG with Mrc1/Tof1/Csm3/Ctf4 and RPA collided with a nucleosome which is partially unwrapped to SHL (–3).

Supplementary Movie 12: The representative trajectory in which the lagging strand associated with the exposed H2A/H2B dimer during the collision between CMG and a nucleosome.

Supplementary Movie 13: The representative trajectory of collisions between CMG and a nucleosome with FACT. The orange beads represent FACT.
